# A new species of genus *Syntactus* Förster (Hymenoptera, Ichneumonidae, Ctenopelmatinae) with a key to Oriental and Eastern Palearctic species

**DOI:** 10.3897/zookeys.170.2321

**Published:** 2012-02-22

**Authors:** Shu-Ping Sun, Mao-Ling Sheng

**Affiliations:** 1 General Station of Forest Pest Management, State Forestry Administration, 58 Huanghe North Street, Shenyang 110034, P. R. China

**Keywords:** Pionini, *Syntactus*, new species, Key, taxonomy, Oriental Region, Palaearctic Region, China

## Abstract

A new species, *Syntactus jiulianicus* Sun & Sheng, **sp.n.** belonging to the tribe Pionini of the subfamily Ctenopelmatinae (Hymenoptera, Ichneumonidae), collected from Jiangxi Province, China, is reported. Illustrations of the new species and *Syntactus delusor* (Linnaeus, 1758), *Syntactus minor* (Holmgren, 1857) and *Syntactus varius* (Holmgren, 1858) are provided. A key to the species of *Syntactus* known from the Oriental and Eastern Palaearctic Regions is given.

## Introduction

*Syntactus* Förster, 1869, belonging to the tribe Pionini of the subfamily Ctenopelmatinae (Hymenoptera, Ichneumonidae), comprises six described species (Horstmann 2007, Kasparyan and Khalaim 2007, Yu et al. 2005), all of them distributed in the Palaearctic Region, three of which are from the Eastern Palearctic.

The European species of *Syntactus* Förster were catalogued by Aubert (2000). Kasparyan and Khalaim (2007) provided a key to the species of the Russian Far East. Two species in Yu et al. (2005), *Syntactus aigneri* (Kiss, 1926) and *Syntactus croaticus* (Kiss, 1926), were subsequently synonymized with, respectively, *Phobetes leptocerus* (Gravenhorst, 1820) and *Phobetes atomator* (Muller, 1776) by Horstmann (2007). One species, *Syntactus delusor* (Linnaeus, 1758), was recorded from Shanxi, China (Chao 1976, Uchida 1952). So far, no species of the genus have been described from the Oriental Region.

In this article a new species belonging to *Syntactus*, collected in Jiangxi Province, situated at the northern border of the Oriental part of China, are reported.

## Materials and methods

Specimens were collected with entomological nets in the forests of Jiulianshan Natural Reserve, Longnan County, Jiangxi Province (CHINA). The forest composed of mixed deciduous angiosperms and evergreen conifers, mainly including *Castanea* spp., *Castanopsis fabri* Hance, *Cinnamomum* spp., *Dalbergia hupeana* Hance, *Dendropanax dentiger* (Harms) Merr., *Machilus* spp., *Quercus* spp., *Tsoongiodendron odorum* Chun, *Pinus massoniana* (Lamb.).

Images of whole bodies were taken using a CANON Power Shot A650 IS. Other images were taken using a Cool SNAP 3CCD attached to a Zeiss Discovery V8 Stereomicroscope and captured with QCapture Pro version 5.1. Specimens of *Syntactus delusor* (Linnaeus, 1758), *Syntactus minor* (Holmgren, 1857), *Syntactus varius* (Holmgren, 1858) and *Syntactus fusiformis* (Thomson, 1894), preserved in Zoologische Staatssammlung München, Germany, (ZSM), were checked.

The morphological terminology is mostly that of Gauld (1991). Wing vein nomenclature is based on Ross (1936) and the terminology on Mason (1986, 1990).

Type specimens are deposited in the Insect Museum, General Station of Forest Pest Management, State Forestry Administration, People’s Republic of China.

## Taxonomy

### Syntactus

Förster, 1869

http://species-id.net/wiki/Syntactus

Syntactus Förster, 1869. Verhandlungen des Naturhistorischen Vereins der Preussischen Rheinlande und Westfalens, 25(1868):210. Type-species: *Ichneumon delusor* Linnaeus; designated by Perkins 1962.
Tromopoea Förster, 1869:210. Type-species: *Catoglyptus minor* Holmgren; designated by Perkins 1962.
Brischkea Kriechbaumer, 1897:167. Type-species: (*Brischkea parvula* Kriechbaumer) = *delusor* Linnaeus; monobasic.

#### Diagnosis.

*Syntactus* can be distinguished from all other genera of Pionini by the combination of the following characters: Apical margin of clypeus blunt. Clypeal foveae open, extraordinarily impressed. Subbasal portion of lower margin of mandible sharp, its outer face without a basal impression. Upper end of epicnemial carina reaching front margin of mesopleuron. Areolet absent. Nervellus subvertical. Glymma absent. Ovipositor thin, needle-like, straight or slightly upcurved.

#### Key to species of Syntactus Förster known in Oriental and Eastern Palaearctic Regions

**Table:** 

1	Mesopleuron without wrinkles, with indistinct or distinct punctures (Figure 3)	2
–	Mesopleuron with distinct, dense and oblique wrinkles	4
2	Face with dense and distinct punctures. Upper tooth of mandible slightly longer than lower tooth	*Syntactus varius* (Holmgren)
–	Face almost smooth and without puncture, or with weak punctures. Upper tooth of mandible evidently shorter than lower tooth (Figure 2a)	3
3	Area superomedia longer than wide (Figure 4), costula connecting in front of its middle. Mesopleuron and mesosternum yellow. Metapleuron reddish brown (female) or yellow (male). Hind legs reddish brown	*Syntactus jiulianicus* Sun & Sheng, sp.n.
–	Area superomedia approximately as long as wide, costula connecting at its middle. Mesopleuron, mesosternum, metapleuron and hind legs black	*Syntactus leleji* Kasparyan
4	Face almost smooth, punctures indistinct. Hind femora reddish brown. (Figure 7)	*Syntactus delusor* (Linnaeus)
–	Face with dense punctures. Hind femora black or brownish black. (Figure 8)	*Syntactus minor* (Holmgren)

### Syntactus jiulianicus

Sun & Sheng sp. n.

urn:lsid:zoobank.org:act:B09BEBC3-C706-48F6-B7B6-2EAF91361025

http://species-id.net/wiki/Syntactus_jiulianicus

Figures 1–6

#### Etymology.

The name of the new species is based on the type locality.

#### Types.

*Holotype*, Female, CHINA: Jiulianshan Natural Reserve, Longnan County, 629 m, Jiangxi Province, 27 April 2011, leg. Mao-Ling Sheng. Paratype: 1 male, same data as holotype.

#### Diagnosis.

*Syntactus jiulianicus* can be distinguished from all other species of *Syntactus* by the combination of the face and clypeus smooth and shining, sparsely and finely punctuate; gena and frons impunctate; face, mesopleuron and mesosternum yellow; first to third terga of female reddish brown, male yellow.

#### Description.

**Female.** Body length approximately 8.5 mm. Fore wing length approximately 7.0 mm. Antenna length approximately 7.5 mm.

**Head.** Face and clypeus almost smooth (Figure 2a). Face approximately 1.9 times as wide as long, with indistinct, uneven and fine punctures; median portion weakly and longitudinally convex. Clypeus gradually raised towards apical margin, with very sparse, fine and distinct punctures. Median portion of mandible with longitudinal wrinkles; upper tooth of mandible evidently shorter than lower tooth. Cheek nearly smooth, without punctures. Malar space approximately 0.6 times as long as basal width of mandible. Gena and vertex (Figure 2) and frons smooth and shining. Gena impunctate, hind portion slightly convergent posteriorly, in lateral view approximately 0.9 times as long as width of eye. Posterior-lateral portion of vertex with indistinct fine punctures, hind-median portion slightly concave. Interocellar area slightly convex. Postocellar line about 0.7 times as long as ocular-ocellar line. Frons impunctate; upper-median portion weakly convex; lower portion slightly concave. Antenna with 37 flagellomeres. Ratio of length from first to fifth flagellomeres: 2.0:1.8:1.4:1.3:1.3. Occipital carina complete, dorsomedian portion concave, lower end reaching base of mandible.

**Figures 1–6.**
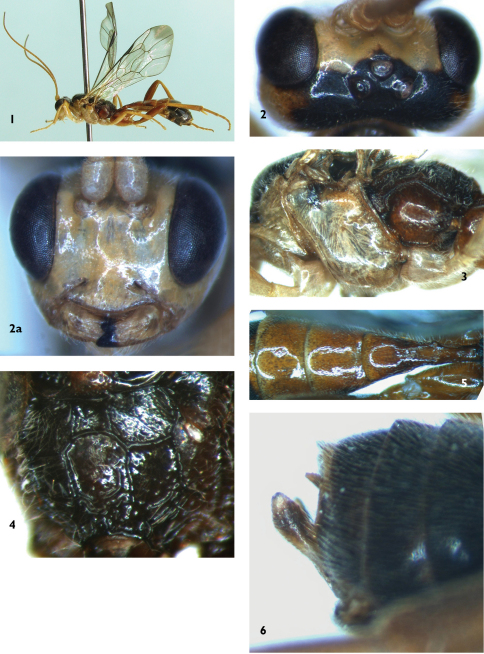
*Syntactus jiulianicus* Sun & Sheng, sp.n. Holotype. Female **1** Body, lateral view **2** Head, dorsal view 2a Head, anterior view **3** Mesosoma, lateral view **4** Propodeum **5** Terga 1 to 3, dorsal view **6** Apical portion of metasoma, lateral view.

**Mesosoma.** Anterior portion of pronotum smooth, lateral concavity with dense oblique fine wrinkles, dorsal-porsterior portion with fine punctures. Epomia present, weak. Mesoscutum with dense and fine punctures. Notauli present, anterior 0.3 sharp. Scutoscutellar groove with weak longitudinal wrinkles. Scutellum convex, with fine punctures, basal 0.3 with lateral carina. Postscutellum weakly convex, approximately quadrate. Subalar prominence strongly convex. Mesopleuron (Figure 3) and metapleuron smooth and shining. Lower portion of mesopleuron with sparse and indistinct fine punctures. Epicnemial carina strong, upper end reaching subalar prominence. Posterior portion of metapleuron with fine oblique wrinkles. Submetapleural carina strongly, anterior portion evidently convex. Wings slightly brownish, hyaline, apical portions smoky-gray. Fore wing with vein 1cu-a distal of 1/M by 0.2 times as long as 1cu-a. Vein 2-Cu approximately 1.8 times as long as 2cu-a. Hind wing vein 1-cu about 1.5 times as long as cu-a. Apical edge of fore tibia with a small tooth at outer side. Hind coxa smooth, with sparse and fine punctures. Ratio of length of hind tarsomeres 1:2:3:4:5 is 5.3:2.7:1.9:1.1:1.4. Propodeum (Figure 4) evenly convex. Area basalis slightly longer than wide, smooth, convergent anteriorly. Area superomedia longer than wide, costula connecting in front of its middle, combined with area petiolaris, combined area smooth, nearby apical margin with distinct transverse fine wrinkles, from costula to apex evidently convergent posteriorly. Area externa with fine punctures and gray hairs. Area lateralis with dense oblique wrinkles. Pleural carina distinctly curved at the level of propodeal spiracle, with carina between pleural carina and propodeal spiracle. Propodeal spiracle approximately 2.6 times as long as wide.

**Metasoma.** Terga almost smooth. First tergum approximately 2.3 times as long as apical width, strongly convergent toward base, median dorsal carinae indistinct. Dorsolateral carinae weak, subbasal portion near spiracle indistinct. Ventrolateral carinae complete. Spiracle convex, located slightly before middle of first tergum. Second tergum trapeziform, approximately 0.7 times as long as apical width. Third tergum approximately 0.5 times as long as apical width. Ovipositor sheath approximately 0.7 times as long as apical depth of metasoma, subapical portion distinctly wider than basal portion (Figure 6). Ovipositor very thin.

**Color.** (Figure 1). Main body and legs yellow, except the following. Flagellum reddish brown. Apical teeth of mandibles, vertex, collar, mesoscutum, lateral portions of scutellum and postscutellum, axillary troughs of mesonotum and metanotum, a small spot beneath subalar prominence, fourth to seventh terga except narrow hind margins black. Median portions of scutellum and postscutellum red. Propodeum darkish brown, lateral portion fuscous. Terga 1 to 3 reddish brown. Posterior-lateral portions of third and fourth terga with longitudinal brownish black spots. Metapleuron and hind legs reddish brown. Stigma blackish brown. Veins dust-colored.

**Male.** Body length approximately 7.0 mm. Fore wing length approximately 5.6 mm. Antenna length approximately 7.0 mm. Antenna with 35 flagellomeres. Ventral profiles of hind coxae, metapleuron, terga 1 to 3 and hind portion of tergum 4 yellow.

**Figure 7.**
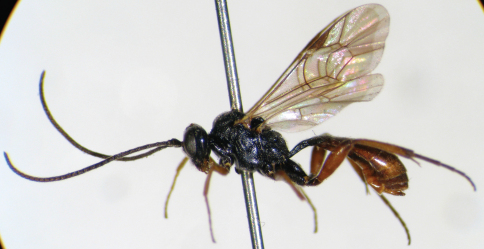
*Syntactus delusor* (Linnaeus, 1758). Female. Body, lateral view.

**Figure 8.**
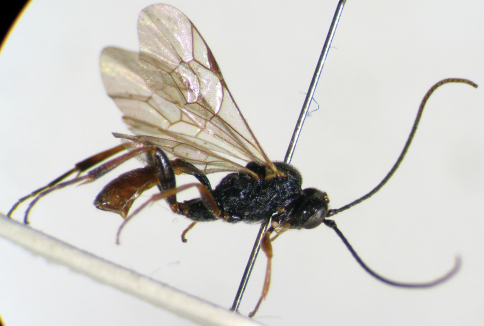
*Syntactus minor* (Holmgren, 1857). Female **8** Body, lateral view.

**Figure 9.**
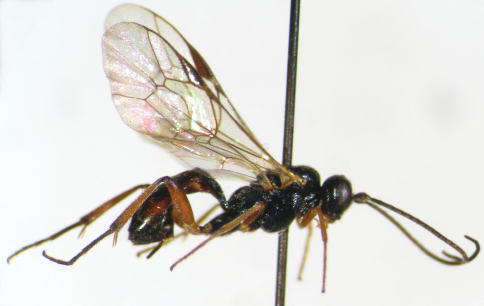
*Syntactus varius* (Holmgren, 1858). Female. Body, lateral view.

## Supplementary Material

XML Treatment for Syntactus

XML Treatment for Syntactus jiulianicus
